# Cellular Senescence of Lens Epithelial Cells and Age-Related Cataract: A Systematic Review

**DOI:** 10.3390/bioengineering13040433

**Published:** 2026-04-07

**Authors:** Anastasia Kourtesa, Konstantinos Skarentzos, Georgios S. Dimtsas, Periklis G. Foukas, Marilita Moschos

**Affiliations:** 1 2nd Department of Pathology, University General Hospital “Attikon”, National and Kapodistrian University of Athens, 12462 Athens, Greece; k.skarentzos@gmail.com (K.S.); pfoukas@yahoo.com (P.G.F.); 2 1st Department of Ophthalmology, General Hospital of Athens “G. Gennimatas”, National and Kapodistrian University of Athens, 11527 Athens, Greece; gdimtsas@gmail.com (G.S.D.); moschosmarilita@yahoo.fr (M.M.)

**Keywords:** cell, senescence, molecular aging, cataract, lens

## Abstract

Recent evidence links lens epithelial cell (LEC) dysfunction and cellular senescence—an irreversible cell cycle arrest with a pro-inflammatory secretory phenotype—to age-related cataract (ARC) progression. This systematic review synthesizes current knowledge on LEC senescence, its molecular features, and laboratory methods for senescence assessment in the ARC. Following PRISMA guidelines, a comprehensive search of PubMed, Scopus and Cochrane databases retrieved 3417 records from inception to 9 February 2025, with 14 studies ultimately included (821 patients and multiple in vitro LEC models). The following multiple senescence expression pathways were identified: SA-β-gal activity, p53/p21 and p16INK4A pathway activation, mitochondrial dysfunction, oxidative stress, and secretion of senescence-associated secretory phenotype (SASP) factors. Notably, cortical cataract demonstrated direct association with local senescent cell accumulation, while nuclear cataract reflected cumulative oxidative damage from impaired LEC-mediated antioxidant defense. Senescence markers correlated positively with cataract severity across multiple studies. Several potential therapeutic targets emerged, including metformin (AMPK activation/autophagic restoration), circMRE11A silencing, NLRP3 inflammasome inhibition, and modulation of FYCO1/PAK1 and MMP2 pathways. This review establishes LEC senescence as a central process in ARC pathogenesis and highlights promising senotherapeutic approaches. Future research should prioritize human surgical samples, develop standardized senescence detection panels (SA-β-gal + p21/p16 + SASP factors), and conduct longitudinal studies to establish causal relationships between senescence accumulation and cataract progression.

## 1. Introduction

Cataract is a leading cause of visual impairment worldwide [1] and remains a significant public health challenge despite being treatable through surgery. Age-related cataract (ARC) constitutes the majority of cases, with age-standardized pooled prevalence estimate (ASPPE) globally at 17.20% [2]. ARC arises from progressive degeneration of the lens, yet the underlying cellular mechanisms remain incompletely understood. The gold standard classification method is the Lens Opacity Classification System III (LOCS III) [2,3]. The most common treatment consists of phacoemulsification [4,5].

The crystalline lens of the eye is a transparent tissue composed of lens epithelial cells (LECs). It is believed that LECs function disturbance led to loss of lens transparency and to cataract formation [6]. A link between cellular senescence in lens epithelial cells and the progression of ARC was suggested [7]. Unlike apoptosis, senescent cells remain alive and metabolically active [8].

A hallmark of senescence is irreversible cell cycle arrest, regulated by p16INK4A and the p53-p21-Retinoblastoma (RB) pathways, and accumulation of senescent cells that form a senescence-associated secretory phenotype (SASP). Intermediate levels of p53 promote the expression of anti-apoptotic Bcl-2 family proteins. Additionally, p21 inhibits caspase-3 and apoptosis, making them apoptosis-resistant and contributing to their accumulation in tissues [9,10]. Senescence is associated with increased lysosomal senescence-associated-galactosidase activity (SA-β-gal) and can also affect the nuclear lamina organization and cause reduction in lamin B1. Senescent cells undergo morphological changes, becoming larger, flatter, and more irregularly shaped [9,10].

Emerging evidence suggests that the impact of LEC senescence may vary according to cataract subtype. Cortical cataract, due to its anatomical proximity to the LEC monolayer, may be more directly influenced by local senescent cell accumulation and SASP-mediated effects on adjacent cortical fibers [11]. In contrast, nuclear cataract, despite affecting the central lens region, may reflect the cumulative consequence of decades of impaired LEC-mediated antioxidant protection and glutathione maintenance [12,13]. Understanding these differential associations could inform subtype-specific therapeutic strategies targeting cellular senescence.

Multiplexing in LEC senescence determination is necessary as the amount and the combination of required markers is still uncertain. The core set of most used senescence markers include: p16, p21, SASP factors (IL-6, IL-8, IL-1β) and SA-β-gal.

This systematic review aims to explore the role of cellular senescence in lens epithelial cells and its impact on the pathophysiology of ARC, in accordance with the laboratory methods of senescence estimation shedding light on a critical aspect of cataract biology that could inform future potential therapeutic strategies.

## 2. Materials and Methods

### 2.1. Study Design and Eligibility Criteria

This systematic review was in accordance with PRISMA (“Preferred-Reporting-Items-for-Systematic-Reviews-and-Meta-Analyses”) guidelines (http://www.prismastatement.org/; accessed on 15 July 2024) and the protocol was registered at PROSPERO (CRD420250649896) on 24 February 2025. PRISMA checklist and PRISMA abstract checklist can be found in Supplementary Figures S1 and S2 respectively [14].

PICO (Population, Intervention, Comparison, Outcome) framework was used. Population consisted of ARC patients and in vitro studies with human cells. Phacoemulsification was acceptable as intervention. Studies with comparisons between human senescent cells in ARC and healthy human LECs were included. Regarding outcomes, senescence markers were essential for inclusion.

Reviews, case reports, case series, and not-peer-reviewed articles were excluded, as well as animal studies (in vivo or in vitro). Articles written in English and German were included. Papers referred to other types of non-ARC or secondary cataract due to certain ocular or systemic diseases (e.g., Down syndrome, glaucoma, arthritis, and diabetes) were eliminated. Acceptable senescence markers followed the Guidelines for minimal information on cellular senescence experimentation in vivo of 2024 [10].

### 2.2. Literature Search Strategy

Two blinded reviewers (A.K. and K.S.) searched PubMed, Scopus, and Cochrane from inception until 9 February 2025. The search algorithm was ((cell AND senescence) OR (molecular aging)) AND ((cataract) OR (lens)). Eligibility criteria were applied in articles’ title and abstract. The remaining articles were reviewed in full text, while reporting exclusion reasons. Consensus was reached via discussion. Snowballing was conducted.

### 2.3. Data Extraction

Two authors performed data extraction independently. Consensus was reached via discussion. Authors’ names, publication year, demographics, cataract surgery, LOCS III classification, control groups, and senescence markers were extracted from all included articles.

### 2.4. Study Selection and Quality Assessment

Two independent authors assessed the quality of included studies via OHAT tool [15] and OHAT tool modified for in vitro studies [16]. This quality assessment tool consists of 9–11 questions. EndNote™ 20 was used as citation manager [17].

## 3. Results

### 3.1. Study Selection

**Figure 1:**
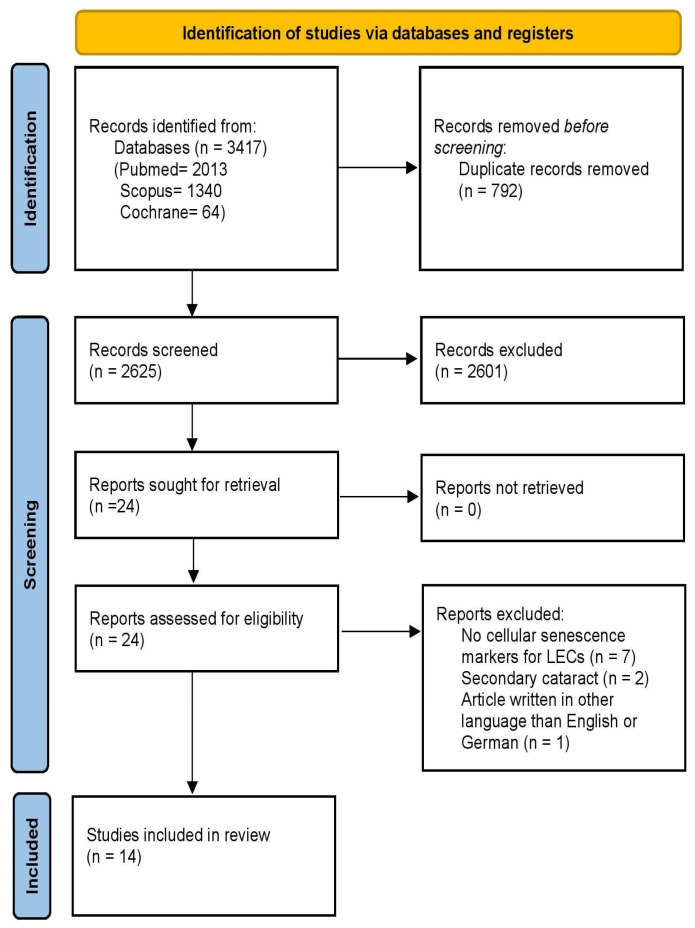
PRISMA 2020 flow diagram.

A total of 3417 records were identified through literature search. Following the exclusion of 792 duplicate records, titles and abstracts of 2625 unique articles were screened for eligibility. Of these, 24 were assessed in full text, and 10 were excluded: seven articles lacked cellular senescence markers (reporting only proliferation or apoptosis markers, or associated senescence markers with apoptosis pathways), two investigated secondary cataract (posterior capsule opacification; a common complication after cataract surgery) and one was written in Chinese. Ultimately, 14 studies met the predetermined inclusion criteria [11,12,13,18,19,20,21,22,23,24,25,26,27,28] and were incorporated into the qualitative data synthesis, as shown in Figure 1.

### 3.2. Study Characteristics

Both human samples extracted from cataract surgery and human origin cell lines [primary human LECs (HLECs), HLE-B3, SRA01/04] [11,19,24,26,28] were studied in 5 of the 14 aforementioned studies [11,12,13,18,19,20,21,22,23,24,25,26,27,28]. Human samples were studied in three of the included articles [12,13,23], while data from the rest eligible records were obtained from in vitro studied cell lines of human origin [18,20,21,22,25,27].

Totally, 821 patients were included in this systematic review. Patients’ and control groups’ demographics are demonstrated on Table 1. Six out of eight authors used LOCS III for cataract classification [11,12,13,19,23,24].

As summarized in Table 2, the included studies utilized a combination of human surgical specimens (*n* = 821 patients across eight studies) and human lens epithelial cell lines, including primary HLECs, HLE-B3, and SRA01/04. Further details regarding methods of senescence estimation are presented in Table 2.

Here we quote a brief description of different cell lines used by eligible studies in this systematic review: Primary HLECs consist of human lens epithelial cells isolated from ARC patients and cultured in Dulbecco’s modified Eagle’s medium (DMEM) containing 15% fetal bovine serum (FBS) [11,29]. HLE-B3 cell line was derived from infant human lens epithelia, and it was cultured in DMEM containing 10% fetal bovine serum with 100 U/mL penicillin and 100 μg/mL streptomycin. Most of the authors used them as described or with some minor adjustments [18,19,21,25,26,27]. SRA01/04 cell lines, from human origin, were cultured in DMEM containing 10% FBS with 100 U/mL penicillin and were used as mentioned or with few adjustments [20,22,24,28].

### 3.3. Risk of Bias

The overall quality of the included studies was moderate to high. Most of the studies were low risk according to the results presented in Table 3, where the overall outcome of all eligible articles is demonstrated. Risk of bias assessment was conducted differently in studies that included patients and human cell lines. Some questions overlap in cohorts or case–control studies with experimental studies. Also, there are questions that are not applicable in experimental studies but are applicable in cohorts or case–control studies and vice versa. Thus, we had to examine the risk of bias in the experimental part of the article and in the cohort or case–control part separately. So, two different scores about risk of bias are demonstrated regarding these studies in Table 3 [11,19,24,26,28]. The dual-assessment approach (separate evaluation of human and experimental components) enhances transparency and allows for nuanced interpretation. Nineteen separate OHAT assessments were conducted (8 human, 11 experimental). The overall quality is favorable, with most domains rated as low risk of bias, though notable patterns emerge across study types and specific domains. Experimental components consistently rated higher (predominantly “++” and “+”) with excellent randomization, allocation concealment, and exposure characterization, so their overall confidence was ranked moderate–high and was primarily limited by lack of blinding, though objective markers mitigate this concern. Human observational components showed greater variability, with particular strengths in confounding control and outcome reporting, but there were some concerns about selection bias and attrition, while the overall human evidence confidence is estimated as moderate (strengthened by excellent confounding control, but limited by selection and attrition concerns).

### 3.4. Results of Individual Studies

#### 3.4.1. Synthesis of Included Studies

The included studies demonstrated considerable heterogeneity in their approach to senescence assessment yet revealed patterns across experimental models and human samples. Table 2 provides a comprehensive summary of all 14 included studies, detailing their design, cellular models, senescence induction methods, molecular markers assessed, and principal findings related to ARC pathophysiology. As shown in Table 2, SA-β-gal activity was the most frequently employed senescence marker, utilized in 12 of 14 studies [11,12,18,19,20,21,22,23,24,26,27,28], followed by p21 expression analysis in 11 studies [12,13,19,20,21,23,24,25,26,27]. This methodological diversity, while reflecting the absence of a standardized senescence detection panel, provides complementary evidence supporting the role of LEC senescence in ARC pathogenesis.

#### 3.4.2. Cohorts and Case–Control Studies

Analysis of human lens epithelial samples from cataract surgery patients revealed upregulation of senescence markers compared to non-cataract controls (Table 2). Huang et al. suggested that senescence in human LECs is strongly associated with a failure in redox regulation, impaired Biliverdin reductase A nuclear trafficking (Biliverdin reductase A has a ROS-scavenging ability), and defective antioxidative responses, all of which contribute to oxidative damage, mitochondrial dysfunction, and premature cellular aging. In addition, it was shown that low levels of Biliverdin reductase A in patients’ eyes induced cataract formation and cellular senescence, especially in nuclear cataract [12]. Similarly, Wang et al. [13] observed significant p21 upregulation in 18 age-related nuclear cataract (ARNC) patients compared to 18 age-matched controls, alongside decreased expression of the protective antioxidant enzyme HO-1.

Another study divided patients into hyperuricemia and normouricemia groups. NLRP3 (nucleotide-binding oligomerization domain-like receptor pyrin domain containing-3) inflammasome activates cytokines in macrophages and its dysregulation has been shown to induce cataract development. According to the study of Lin et al., patients with hyperuricemia showed higher activation of the NLRP3 inflammasome, with a notable increase in cellular senescence, as indicated by the higher number of SA-β-gal-positive cells, compared to normouremic patients. These results suggest a direct link between elevated uric acid in the aqueous humor, NLRP3 inflammasome activation and cellular aging of LECs in the progression of cataract [23].

#### 3.4.3. Cohort or Case–Control Studies with Experimental Part (Human Cell Lines)

Chen et al. [19] demonstrated that human lens epithelium from ARC patients exhibited morphological signs of senescence (flattened, sparse cells) with elevated SA-β-gal staining and increased expression of p53 and p21 compared to controls. In vitro, H_2_O_2_-treated HLE-B3 cells showed similar morphological changes, increased SA-β-gal activity, and upregulation of senescence-associated genes (p53, p21, p16, IL-6, and IL-8), reinforcing oxidative stress as a key driver of LEC senescence and cataractogenesis [19].

The research of Fu et al. [11] points out an age-related decline in lens stem cells (LSCs), with abundant Ki67+ cells in children (0–10 years) but few in adults over 60. LECs from older individuals lost proliferative activity, exhibited senescent morphology, and showed increased SA-β-gal staining. Cortical cataract severity correlated strongly with the percentage of SA-β-gal+ LECs, implicating LEC senescence in cortical cataract formation [11].

Liu et al. [24] also identified cortical cataract as demonstrating the strongest correlation with senescence markers (SA-β-gal positivity, p53/p21 upregulation). They identified circMRE11A as a key regulator: its silencing increased LEC viability and promoted cell cycle progression, while its overexpression induced cell cycle arrest and reduced viability, suggesting a role in stress-induced LEC aging [24].

Also, Yan et al. [26] indicated that p53 levels increased with cataract severity (LOCS III grade), while p21 showed no significant change. Total laminins (LMs) and LMα4 were elevated in anterior lens capsules of higher-grade cataracts. Specifically, in grade V cataractous ALCs, LMα4 was significantly higher than that in grade II ALCs. In H_2_O_2_-treated HLE B-3 cells, senescent morphology, increased SA-β-gal activity, elevated LMs, and p53/p21 overexpression were observed, suggesting excessive laminin deposition in senescent LECs contributes to ARC progression [26].

According to another study, N-myc downstream-regulated gene 2 (NDRG2) expression was upregulated in cells with H_2_O_2_ treatment and in patients with cataractous lenses compared to clear lenses. So, overexpression of NDRG2 was linked to an increased sensitivity of cells to oxidative injury. Both mRNA and protein levels of NDRG2 were upregulated in cataract tissues, suggesting a potential association between NDRG2 expression and the progression of ARC, which was enhanced by the SA-β-gal staining results in patient’s LECs and in vitro in cultured HLECs (SRA01/04). Upregulation of NDRG2 seems to induce cell morphological changes and reduces cellular resistance to oxidative stress, which can lead to cellular senescence and ARC formation [28].

#### 3.4.4. Experimental Studies (Human Cell Lines Only)

Ahmadi et al. [18] demonstrated that low-dose ionizing radiation induces DNA damage in HLE-B3 and primary HLECs, with increased ROS production and persistent DNA breaks up to 24 h. Although telomere length, telomerase activity, and γ-H2AX foci showed no significant changes, a dose-dependent increase in SA-β-gal-positive cells was observed over time, indicating premature senescence and implicating radiation-induced senescence in cataract formation [18].

Chen et al. [20] showed that FYCO1 downregulation in H_2_O_2_- or UVB-treated SRA01/04 cells reduced autophagic flux and increased p21 expression. Conversely, FYCO1 knockout attenuated senescence, as evidenced by reduced SA-β-gal staining and decreased PAK1 expression. Suppression of PAK1 in FYCO1-knockout cells further limited senescence, identifying the FYCO1/PAK1 axis as a potential therapeutic target in cataract [20].

The results of Deng et al. [21] study demonstrated that MMP2 interference via siRNA in H_2_O_2_-treated HLE-B3 cells significantly reduced DNA damage and cellular senescence. Compared to controls, the H_2_O_2_+siRNA-MMP2 group showed decreased SA-β-gal staining and reduced expression of senescence markers γ-H2AX, p16, and p21, indicating that MMP2 inhibition protects against oxidative stress-induced LEC senescence.

Li et al. [22] highlight that H_2_O_2_ treatment of SRA01/04 cells induced a dose-dependent increase in SA-β-gal-positive cells and senescent morphology, accompanied by downregulation of senescence marker protein 30 (SMP30), an antioxidant and antiapoptotic protein. These findings suggest that persistent oxidative stress promotes LEC senescence through SMP30 depletion, contributing to cataractogenesis [22].

The study of Seomun et al. [25] showed that sublethal H_2_O_2_ exposure in HLE B-3 cells induced G2/M cell cycle arrest without apoptosis, mediated by p21 accumulation. p21 inhibition reduced G2/M arrest, confirming that oxidative stress drives LEC senescence through p21-dependent cell cycle disruption, further linking senescence to cataract formation.

The expression of a series of age-related markers (p53, p21, p16 and the inflammatory cytokines IL-6 and IL-8) was according to Zhang et al. increased in the H_2_O_2_-induced senescence model in HLE-B3 cells. Chen et al. [19] and Zhang et al. [27] both demonstrated that metformin treatment attenuated H_2_O_2_-induced senescence through AMPK activation and autophagic flux restoration, suggesting a potential pharmacological approach to delaying cataract progression.

### 3.5. Findings from In Vitro Studies

Across multiple studies using HLE-B3 [18,19,21,25,26,27] and SRA01/04 cell lines [20,22,24,28], H_2_O_2_ exposure induced: increased SA-β-gal activity [11,12,18,19,20,21,22,23,24,26,27,28], upregulation of p53/p21 pathway components [12,13,19,20,21,23,24,25,26,27], elevated SASP factor expression (IL-6, IL-8) [19,23,27] and morphological changes characteristic of senescence [11,19,26].

### 3.6. Correlation with Cataract Type/Stage and Severity

A critical finding emerging from Table 2 is the relationship between senescence marker accumulation and cataract severity. Six studies employing LOCS III classification [11,12,13,19,23,24] reported positive correlations between senescence markers and cataract grade. Most notably, Yan et al. [26] demonstrated progressive increases in p53 expression and laminin deposition correlating with cataract severity from grade II to V, providing compelling evidence that LEC senescence contributes to disease progression rather than representing an epiphenomenon.

The association between cataract subtype and senescence markers merits particular attention. Fu et al. [11] and Liu et al. [24] both identified cortical cataract as demonstrating the strongest correlation with senescence markers, including SA-β-gal positivity and p53/p21 upregulation. In contrast, nuclear cataract specimens showed distinct molecular signatures, with Huang et al. [12] reporting decreased BVRA expression and Wang et al. [13] identifying impaired autophagic flux as characteristic features.

## 4. Discussion

ARC is a really common eye disease, but the exact pathogenetic mechanism is not yet fully understood. The results of our systematic review indicate that cellular senescence of LECs plays a crucial role in the lens opacification and finally in the cataract formation.

In order to create in vitro models of aging, the most common inducer of premature senescence-like phenotype in cells is H_2_O_2_, based on the alterations in the senescence pathway from the oxidative stress [30,31,32,33]. Since premature senescence induced by extended exposure to oxidative stress displays similar mechanisms with pathological aging in vivo, it can serve as a useful model of studying aging in vitro [34]. UVB radiation can also be used to intrigue senescence in cells, but it is not so commonly used, probably because it is a more complicated, time-consuming method and requires special laboratory equipment [18].

As per our analysis, the most commonly used senescence inducers are H_2_O_2_ and UVB radiation, with H_2_O_2_ being far more used than the radiation technique. When it comes to comparison of responses of different cell lines to the same stressor, in vitro study of senescence presents many advantages, such as the ability to use many different laboratory techniques; the studied cells can be of large numbers and easier to be obtained and preserved, but it underestimates the complexity of induced aging by focusing exclusively on cellular damage mediated by oxidative stress. Thus, we insist on the use of human samples extracted from cataract surgeries, because they will represent more accurately the complexity of mechanisms that form the final senescent cells and lead us to a better understanding of ARC pathophysiology.

After thorough search of the existing literature, we reported the applied methods of senescence estimation, which present great heterogeneity among different authors. The most commonly used senescence estimation method in the vast majority of the studies was SA-β-gal assay [11,12,18,19,20,21,22,23,24,26,27,28], followed by the detection of p21 positive cells through IHC, WBA, IF or qRT-PCR [12,13,19,20,21,24,25,26,27]. Similar techniques were also used either for the detection or to quantify the expression of p53 and p16-positive cells [19,23,24,26,27]. Senescent cells do not display a single molecular and cellular change specific to senescence, but rather a phenotype that includes the cell itself and its microenvironment. Thus, the nature of senescent cells and the absence of a well-defined and globally recognized biomarker demands that multiple endpoints should be assessed. The establishment of a senescence estimation laboratory panel would be useful for future authors, in order to achieve homogeneity between the techniques used and make the results comparable.

Some of the researchers reported correlation between the different types of ARCs and expressed senescence markers. Fu et al. and Liu et al. showed that age-related cortical cataract was associated with increased SA-β-gal expression, as well as with increased p53 and p21 expression [11,24]. Moreover, two more study groups correlated the nuclear type of cataract with increase in p21 positive cells and decrease in the concentration of mRNA levels of BVRA and BR, respectively [12,13]. According to the results of our review, a number of studies highlighted the fact that the severity of cataract is correlated with cellular senescence [11,12,13,19,23,24]. In addition, Yan et al. provided data showing that senescence markers (p53, LMs) increase in parallel with the severity of cataract, according to the LCOSIII classification system of cataract [26]. An intriguing finding emerging from this systematic review is the differential association between LEC senescence and specific cataract subtypes (Table 2). Cortical cataract demonstrated direct correlation with senescence markers across multiple studies [11,24,28]. This can be attributed to the anatomical proximity of cortical fibers to the LEC monolayer, making them directly susceptible to SASP factors and disrupted fiber differentiation from senescent progenitors in the germinative zone [11]. Fu et al. [11] and Liu et al. [24] both reported increased SA-β-gal positivity and p53/p21 expression specifically in cortical cataract specimens, while Zhang et al. [28] linked NDRG2-mediated oxidative stress susceptibility to cortical opacification. Nuclear cataract, despite affecting the central lens region remote from the LEC layer, also shows significant associations with LEC senescence through indirect mechanisms [12,13]. The lens nucleus, composed of the oldest fiber cells with minimal protein turnover, depends entirely on the antioxidant protection established and maintained by LECs [35,36]. Senescent LECs exhibit impaired glutathione (GSH) production and reduced expression of protective enzymes such as BVRA and HO-1, leading to cumulative oxidative damage that manifests first in the nucleus [12,13]. Huang et al. [12] specifically demonstrated that BVRA deficiency correlated with nuclear cataract formation, while Wang et al. [13] identified HO-1 downregulation in ARNC patients. Thus, while cortical cataract may reflect direct local effects of LEC senescence, nuclear cataract likely represents the cumulative consequence of decades of compromised LEC-mediated lens maintenance [35]. The study of Lin et al. focused on the role of NLRP3 inflammasome in cataract formation. They showed that increased levels of uric acid with increased activation of NLRP3 inflammasome, which promotes cytokines production, correlate with lens opacification. Among the hyperuremic and normouremic patients no differences were observed regarding cataract grade [23].

Some limitations of the included articles need to be addressed. First of all, due to the heterogeneity of the eligible studies, it was impossible to perform a meta-analysis. Moreover, the number of human candidates in the phacoemulsification cataract extraction procedures was relatively low and the number of studies that used exclusively human samples were only three. Thus, we were forced to include experimental studies, in order to increase our perspective about the relation between cellular senescence and ARC. While cell models can be used for basic research, they often fail to accurately simulate human physiology, as they present restrictions, like the fact that they are not blinded. On the other hand, they are useful in demonstration of pathophysiology mechanisms in a laboratory-controlled environment.

Regarding the limitations of our systematic review, lack of studies with human samples forced us to also accept studies with cell lines as we mentioned before. Nevertheless, the studies we examined were of high quality and moderate-to-low risk of bias. However, age mismatch across studies represents a significant confounding factor. Fu et al. [11] utilized a control group consisting of pediatric patients aged 0–10 years, which, while demonstrating the presence of lens stem cells in young lenses, may not represent an appropriate age-matched comparison for evaluating senescence in age-related pathology. Similarly, Zhang et al. [28] included a control group that was not adequately age-matched to the ARC cohort (46.80 ± 7.4 years vs. 66.00 ± 8.6 years), potentially underestimating baseline senescence levels in controls. Furthermore, three studies [19,23,26] lacked any control group entirely, relying exclusively on in vitro validation or within-cohort comparisons, which limits the robustness of their findings regarding senescence marker elevation in ARC patients. Also, a major limitation is the absence of blinding declaration, regarding the experimental studies [11,18,19,20,21,22,24,25,26,27,28]. The consistent failure to report or implement blinding of research personnel during experiments is a systematic limitation across the experimental literature that should be addressed in future studies. Some of the included studies reported results about animal experiments [12,13,20,23,24,25,26]. Based on our protocol, we did not mention any data related to experiments conducted on animals.

More research is needed in order to establish the pathways which lead to cellular senescence. As previously referred, cellular senescence is not fully understood, and the fact that senescence is characterized by a phenotype rather than a specific characteristic could make such studies complex and heterogeneous. The lack of a globally established laboratory assays for its detection can also complicate the outcomes from different researchers.

The present systematic review aligns with and extends recent scholarly work on cellular senescence in ocular aging. Several comprehensive reviews have recently addressed this topic from different perspectives. Wu et al. [37] conducted a broad review on senescence across multiple ophthalmic diseases, including age-related macular degeneration, diabetic retinopathy, glaucoma, cataracts, and ocular surface disorders, highlighting the therapeutic potential of senolytics, senomorphics, and epigenetic reprogramming strategies. Their study is the first to systematically integrate the multifaceted mechanisms of cellular senescence across ocular diseases, revealing differential regulatory mechanisms of specific signaling pathways in various ocular pathologies. Similarly, Qin et al. [38] published a detailed narrative review examining the dual role of senescence in lens epithelial cell function and cataractogenesis, with particular emphasis on molecular mechanisms including oxidative stress, mitochondrial dysfunction, and the SASP. Their work provides an excellent mechanistic overview of senescence pathways (p53/p21 and p16/Rb) and discusses emerging senotherapeutic interventions such as dasatinib, quercetin, and metformin. Soleimani et al. [39] provided a narrative review examining the role of senescence in mediating various ophthalmic conditions, with particular attention to corneal pathologies, glaucoma, cataract, and retinal diseases, while also presenting senolytic agents tested in ocular models.

While these reviews offer valuable mechanistic insights and broad overviews across ophthalmology, the present manuscript distinguishes itself through the following several key features: (1) exclusive focus on lens epithelial cell senescence in age-related cataract, providing a depth of analysis not possible in broader multi-disease reviews; (2) adherence to a systematic review methodology with PRISMA guidelines and PROSPERO registration (CRD420250649896), ensuring comprehensive and reproducible literature synthesis; (3) strict application of the 2024 Guidelines for minimal information on cellular senescence experimentation in vivo [10], guaranteeing that only studies employing validated senescence markers were included—a methodological rigor absent from prior narrative reviews; (4) dual assessment of both human surgical specimens and experimental models using distinct OHAT tools for nuanced risk of bias evaluation, enabling quantitative quality appraisal of the evidence base; (5) systematic exploration of differential associations between cataract subtypes (cortical versus nuclear); and (6) identification of a comprehensive panel of potential therapeutic targets specifically for cataract prevention, including metformin, circMRE11A silencing, NLRP3 inflammasome inhibition, and modulation of FYCO1/PAK1 and MMP2 pathways. Thus, while the existing literature establishes the broad mechanistic landscape of cellular senescence in ocular diseases, this systematic review provides the first methodologically rigorous, evidence-based synthesis focused specifically on LEC senescence in ARC, offering actionable recommendations for standardized senescence detection panels and future research directions tailored to cataract research and therapy.

This systematic review possesses several notable strengths that enhance its contribution to the field. First, to our knowledge, this is the first systematic review to comprehensively synthesize evidence on LEC senescence in ARC pathogenesis while adhering strictly to the 2024 Guidelines for minimal information on cellular senescence experimentation in vivo [10], ensuring that only studies employing validated senescence markers were included. Second, the dual assessment of both human surgical specimens and experimental models provided complementary perspectives—human samples offered authentic pathological insights, while in vitro studies enabled mechanistic exploration of senescence pathways and potential therapeutic interventions. Third, the application of distinct OHAT tools for human and experimental studies allowed for nuanced risk of bias assessment, acknowledging the methodological differences between these study types while maintaining rigorous quality standards. Fourth, the correlation between senescence markers and cataract severity across multiple studies [11,12,13,19,23,24,26] strengthens the evidence for a causal relationship rather than mere association.

The clinical value of these findings is substantial. Establishing LEC senescence as a central mechanism in ARC pathogenesis opens new therapeutic avenues beyond surgical intervention, which remains the only available treatment despite its costs and accessibility limitations. The identification of multiple druggable targets—including metformin for AMPK activation and autophagic restoration [19,27], circMRE11A silencing strategies [24], NLRP3 inflammasome inhibitors [23], and modulators of FYCO1/PAK1 [20] and MMP2 pathways [21]—provides a foundation for developing senolytic or senomorphic therapies. Topical therapeutic targeting of circMRE11A could lead to reduction in LECs senescence degree, promoting the cell cycle. This could be achieved using small interfering RNAs (siRNAs) designed to target the unique back-splice junction of Exon 6 and Exon 7, which are unique to the circularized form without affecting the expression of the linear MRE11A mRNA, and for the targeted knockdown the use of transfection reagents to deliver junction-specific siRNAs into LECs could be a recommendation. Another promising strategy is the stimulation of the autophagy pathway through future drugs, focusing on clearing of damaged proteins, which aggregate in the absence of functional FYCO1, as well as targeting the PAX1/p21 axis, as it mediates autophagy and senescence in LECs under oxidative stress. Moreover, topical and/or oral administration of a NLRP3 inflammasome blocker may lead us a step closer to targeted ARC therapy. Interference in inflammatory pathways with blockers may be effective against general inflammation. Furthermore, the differential association of cortical versus nuclear cataract with distinct senescence mechanisms [11,12,13,24,28] suggests that future pharmacological approaches may need to be subtype-specific, potentially enabling personalized prevention strategies, but the impact of senescent cells in the formation of each ARC type (cortical, nuclear, and subcapsular) needs further investigation. The correlation between senescence burden and cataract severity also raises the possibility of using LEC senescence markers as prognostic biomarkers to identify high-risk individuals or monitor disease progression. Ultimately, translating these findings into clinical practice could delay cataract onset, reduce the global burden of cataract blindness, and offer non-surgical options to patients who are poor surgical candidates or lack access to surgical services.

The present systematic review contributes to a better insight into the pathophysiology of cataract formation based on the senescence effect of LECs. The acquired data can also be used in the future for appliance in pharmaceutical and therapeutic measures, with ultimate purpose being the prevention or the delayed onset of cataract. Last but not least, the need for conducting more studies with human samples is deemed essential, in order to establish the effect of cellular senescence (estimated with an accurate and universally approved panel) in the cataract formation and to further explore mechanisms that can interfere with the aging process, making the senolytic drugs a future choice in our therapeutic arsenal for the treatment of ARC.

## 5. Conclusions

The outcomes shed light on the different pathways of cellular senescence and possible pharmaceutical targets like metformin. Obviously, the research about this field is at the very beginning. Nevertheless, it seems that an association exists between cellular senescence and the pathophysiology of ARC. It appears that different types of ARCs are affected more from cellular senescence (nuclear and cortical) than others. Promising data connected ARC severity with increased cellular senescence. It is recommended to prioritize the use of human surgical samples and explore the development of more physiologically relevant models, such as organoids. This approach will also help to establish a standardized “LEC Senescence Detection Panel” which would be multifactorial and may include a combination of laboratory methods (e.g., SA-β-gal + p21/p16 + at least one SASP factor). There is a need for longitudinal studies to establish a causal relationship between the accumulation of senescence markers and cataract progression, as the existing results are relying solely on cross-sectional associations. More research into the pathways and molecules involved in the pathogenetic pathway of cellular senescence-related ARC may indicate new pharmaceutical targets about this very common eye disease of the elderly.

## Figures and Tables

**Table 1:** Demographics of patients and control groups.

Authors (Year of Publication)	LOCS III (Y/N)	Patients (*n*)	Gender (M/F)	Patients’ Age in Years [Mean ± SD/Median (Range)]	Control Group (*n*)	Control Group’s Age in Years [Mean ± SD/Median (Range)]
Chen et al. (2021) [19]	Y	64	NR	NR (50–55)	8	NR
Fu et al. (2016) [11]	Y	190	58/132	NR (50–102)	10	NR (0–10)
Huang et al. (2022) [12]	Y	45	21/24	59.40 ± 4.3	15	57.20 ± 3.90
Lin et al. (2024) [23]	Y	14	8/6	65.54 ± 5.88	N/A	N/A
Liu et al. (2021) [24]	Y	30	14/16	67.70 ± 5.37/68.5 (61–78)	10	60.60 ± 4.97
Wang et al. (2023) [13]	Y	18	10/8	62.67 ± 4.03	18	63.94 ± 3.84
Yan et al. (2019) [26]	N	300	NR	NR (50–97)	N/A	N/A
Zhang et al. (2011) [28]	N	160	81/79	66.00 ± 8.6	59	46.80 ± 7.4

*n* = number; LOCS III = Lens Opacity Classification System III; Y/N = yes/no; M/F = males/females; NR = not reported; N/A = not applicable; SD = standard deviation.

**Table 2:** Summary of included studies on cellular senescence in age-related cataract.

Authors (Year)	Study Design	Sample/Cell Type	Senescence Inducer	Senescence Markers Assessed	Key Findings	Cataract Type/Severity Association
Ahmadi et al. (2022) [18]	Experimental	HLE-B3, primary HLECs	Low-dose ionizing radiation	SA-β-gal, telomere length (q-PCR), telomerase activity, γ-H2AX (IF)	Dose-dependent increase in SA-β-gal+ cells; persistent DNA damage up to 24h; genomic instability observed	Not specified
Chen et al. (2021) [19]	Case–control + Experimental	ARC patients (*n* = 64), HLE-B3	H_2_O_2_	SA-β-gal, p21 (IHC, WBA, qRT-PCR), p53 (IHC, WBA, qRT-PCR), IL-6 (qRT-PCR), IL-8 (qRT-PCR), p-AMPKα (IHC, WBA), p-ACC (IHC, WBA), morphological changes	↑ SA-β-gal in ARC samples; ↑ p53/p21 expression; Metformin restored autophagic flux via AMPK activation; MET alleviated senescence	Severity correlation reported (LOCS III)
Chen et al. (2024) [20]	Experimental	SRA01/04	H_2_O_2_, UVB	SA-β-gal, p21 (WBA, qRT-PCR)	FYCO1 downregulation ↑ senescence markers; FYCO1 knockout ↓ SA-β-gal and p21; PAK1 identified as mediator	Not specified
Deng et al. (2024) [21]	Experimental	HLE-B3	H_2_O_2_	SA-β-gal, p16 (WBA), p21 (WBA), γ-H2AX (IF, WBA)	MMP2 interference ↓ DNA damage and cellular senescence; ↓ γ-H2AX, p16, p21 in H_2_O_2_+siRNA-MMP2 group	Not specified
Fu et al. (2016) [11]	Case–control + Experimental	ARC patients (*n* = 190), primary HLECs	Natural aging	SA-β-gal, Ki-67(IF), morphological changes	↓ Ki-67+ cells with age; ↑ SA-β-gal+ cells in cortical cataract; LSC exhaustion correlated with senescence	Cortical cataract strongly associated with SA-β-gal+ LECs (LOCS III)
Huang et al. (2022) [12]	Case–control	ARC patients (*n* = 45), controls (*n* = 15)	Natural aging	SA-β-gal, p16 (WBA), p21 (WBA)	↓ BVRA levels in ARC; BVRA deficiency ↑ oxidative damage and senescence; impaired redox regulation	Nuclear cataract associated with ↓ BVRA/BR (LOCS III)
Li et al. (2017) [22]	Experimental	SRA01/04	H_2_O_2_	SA-β-gal	Dose-dependent ↑ in SA-β-gal+ cells; ↓ SMP30 expression with H_2_O_2_ concentration	Not specified
Lin et al. (2024) [23]	Case–control	ARC patients (*n* = 14)	Uric acid/NLRP3	SA-β-gal, p21 (WBA), p53 (WBA), IL-1β (WBA, IF)	Hyperuricemia ↑ NLRP3 activation; ↑ SA-β-gal+ cells in hyperuricemic patients; uric acid drives senescence	No difference in cataract grade between groups (LOCS III)
Liu et al. (2021) [24]	Case–control + Experimental	ARCC patients (*n* = 30), controls (*n* = 10), SRA01/04	H_2_O_2_	SA-β-gal, p21 (WBA, IF), p53 (WBA, IF)	ARCC; ↑ SA-β-gal, p53, p21; circMRE11A silencing ↑ cell viability and cycle progression	Cortical cataract (LOCS III); severity correlation reported
Seomun et al. (2005) [25]	Experimental	HLE B-3	H_2_O_2_	p21 (WBA)	H_2_O_2_ induced G2/M arrest via p21 accumulation; no apoptosis; p21 inhibition ↓ G2/M arrest	Not specified
Wang et al. (2023) [13]	Case–control	ARNC patients (*n* = 18), controls (*n* = 18)	Natural aging	p21 (WBA)	↑ p21 in ARNC vs. controls; ↓ HO-1 expression; impaired autophagic flux; HO-1 protects against senescence	Nuclear cataract (LOCS III); severity correlation reported
Yan et al. (2019) [26]	Case–control + Experimental	ARC patients (*n* = 300), HLE B-3	H_2_O_2_	SA-β-gal, p21 (WBA, IF), p53 (WBA), SASP, LMα4 (ELISA, IF, WBA), LMs (IHC)	↑ p53 with age/ARC grade; ↑ LMs in cataractous ALCs; LMα4 highest in grade V; H_2_O_2_ induced morphological changes, ↑ SA-β-gal, ↑ LMs, ↑ p53/p21	Senescence markers increase with cataract severity (LOCS III grades II-V)
Zhang et al. (2020) [27]	Experimental	HLE-B3	H_2_O_2_	SA-β-gal, p21 (WBA, qRT-PCR), p53 (WBA), p16 (qRT-PCR), IL-6 (qRT-PCR), IL-8 (qRT-PCR), p-AMPK (WBA), p-ACC (WBA)	↑ SA-β-gal, p53, p21, p16, IL-6, IL-8 in H_2_O_2_ model; Metformin prevented H_2_O_2_-induced senescence	Not specified
Zhang et al. (2011) [28]	Case–control + Experimental	ARC patients (*n* = 160), controls (*n* = 59), SRA01/04	H_2_O_2_	SA-β-gal	NDRG2 upregulated in cataract tissues and H_2_O_2_-treated cells; NDRG2 overexpression ↑ sensitivity to oxidative stress; ↑ SA-β-gal+ cells	Cortical ARC association suggested

Abbreviations: SA-β-gal: senescence-associated-beta-galactosidase; WBA: Western blot analysis; IHC: immunohistochemistry; IF: immunofluorescence; qRT-PCR: quantitative real-time polymerase chain reaction; q-PCR: quantitative polymerase chain reaction; ELISA: enzyme-linked immunosorbent assay; IL: interleukin; SASP: senescence-associated secretory phenotype; LMs: laminins; LMα4: laminin subunit alpha-4; p-AMPK: phosphorylated-AMPK; p-ACC: phosphorylated-acetyl-CoA carboxylase; γ-H2AX: gamma-H2A histone family member X; HO-1: heme oxygenase-1; BVRA: biliverdin reductase A; BR: biliverdin reductase; NDRG2: N-myc downstream-regulated gene 2; FYCO1: FYVE and coiled-coil domain containing 1; MMP2: matrix metalloproteinase 2; NLRP3: NLR family pyrin domain containing 3; LSC: lens stem cells; ARC: age-related cataract; ARCC: age-related cortical cataract; ARNC: age-related nuclear cataract; HLECs: human lens epithelial cells; LOCS III: Lens Opacity Classification System III; MET: metformin; H_2_O_2_: hydrogen peroxide; ↑: increased; ↓: decreased.

**Table 3:** Quality assessment using OHAT tool (OHAT risk of bias tool for human and animal studies modified for in vitro studies).

Authors	Type of Study	Q1	Q2	Q3	Q4	Q5	Q6	Q7	Q8	Q9	Q10	Q11
Ahmadi et al. (2022) [18]	E	++	++	NA	NA	++	-	+	++	+	+	+
Chen et al. (2021) [19]	H	NA	NA	-	+	NA	NA	+	+	+	++	++
Chen et al. (2021) [19]	E	++	++	NA	NA	++	-	+	++	+	+	++
Chen et al. (2024) [20]	E	++	++	NA	NA	++	-	+	++	+	+	++
Deng et al. (2024) [21]	E	++	++	NA	NA	++	-	+	++	+	+	++
Fu et al. (2016) [11]	H	NA	NA	-	++	NA	NA	+	+	+	++	+
Fu et al. (2016) [11]	E	++	++	NA	NA	++	-	+	++	+	+	+
Huang et al. (2022) [12]	H	NA	NA	++	-	NA	NA	++	+	+	++	++
Li et al. (2017) [22]	E	++	++	NA	NA	++	-	+	++	+	+	+
Lin et al. (2024) [23]	H	NA	NA	-	+	NA	NA	++	+	++	++	++
Liu et al. (2021) [24]	H	NA	NA	++	++	NA	NA	+	+	+	++	++
Liu et al. (2021) [24]	E	++	++	NA	NA	++	-	+	++	+	+	++
Seomun et al. (2005) [25]	E	++	++	NA	NA	++	-	+	++	+	+	+
Wang et al. (2023) [13]	H	NA	NA	++	++	NA	NA	++	+	+	++	+
Yan et al. (2019) [26]	H	NA	NA	-	++	NA	NA	+	+	+	++	++
Yan et al. (2019) [26]	E	++	++	NA	NA	++	-	+	++	+	+	++
Zhang et al. (2011) [28]	H	NA	NA	-	+	NA	NA	+	+	+	++	+
Zhang et al. (2011) [28]	E	++	++	NA	NA	++	-	+	++	+	+	+
Zhang et al. (2020) [27]	E	++	++	NA	NA	++	-	+	++	+	+	++

‘’E’’ (experimental), “H” (human), “++” (definitely low), “+” (probably low), “-“ (not reported/probably high) and “--“ (definitely high), “NA”(not applicable); Q1: Was administered dose or exposure level adequately randomized? Q2: Was allocation to study groups adequately concealed? Q3: Did selection of study participants result in the appropriate comparison groups? Q4: Did study design or analysis account for important confounding and modifying variables? Q5: Were experimental conditions identical across study groups? Q6: Were research personnel blinded to the study group during the study? Q7: Were outcome data complete without attrition or exclusion from analysis? Q8: Can we be confident in the exposure characterization? Q9: Can we be confident in the outcome assessment (including blinding of assessors)? Q10: Were all measured outcomes reported? Q11: Were there no other potential threats to internal validity?

## Data Availability

No new data were created or analyzed in this study. The data presented in this study are available on request from the corresponding author.

## Supplementary Materials

The following supporting information can be downloaded at https://www.mdpi.com/article/10.3390/bioengineering13040433/s1, Figure S1: PRISMA 2020 Checklist, Figure S2: PRISMA 2020 Abstract Checklist. Ref. [14].
